# NEDD4‐induced degradative ubiquitination of phosphatidylinositol 4‐phosphate 5‐kinase α and its implication in breast cancer cell proliferation

**DOI:** 10.1111/jcmm.13689

**Published:** 2018-05-30

**Authors:** Mai Hoang Tran, Eunjeong Seo, Soohong Min, Quynh‐Anh T. Nguyen, Juyong Choi, Uk‐Jin Lee, Soon‐Sun Hong, Hyuk Kang, Alka Mansukhani, Ilo Jou, Sang Yoon Lee

**Affiliations:** ^1^ Department of Biomedical Sciences Chronic Inflammatory Disease Research Center Ajou University School of Medicine Suwon Korea; ^2^ School of Biological Sciences Institute of Molecular Biology and Genetics Seoul National University Seoul Korea; ^3^ Department of Biomedical Sciences Inha University College of Medicine Incheon Korea; ^4^ Department of Chemistry Ajou University Suwon Korea; ^5^ Department of Microbiology New York University School of Medicine New York NY USA

**Keywords:** breast cancer, degradation, NEDD4, PI3K/Akt, PIP2, PIP5Kα, ubiquitination

## Abstract

Phosphatidylinositol 4‐phosphate 5‐kinase (PIP5K) family members generate phosphatidylinositol 4,5‐bisphosphate (PIP2), a critical lipid regulator of diverse physiological processes. The PIP5K‐dependent PIP2 generation can also act upstream of the oncogenic phosphatidylinositol 3‐kinase (PI3K)/Akt pathway. Many studies have demonstrated various mechanisms of spatiotemporal regulation of PIP5K catalytic activity. However, there are few studies on regulation of PIP5K protein stability. Here, we examined potential regulation of PIP5Kα, a PIP5K isoform, via ubiquitin‐proteasome system, and its implication for breast cancer. Our results showed that the ubiquitin ligase NEDD4 (neural precursor cell expressed, developmentally down‐regulated gene 4) mediated ubiquitination and proteasomal degradation of PIP5Kα, consequently reducing plasma membrane PIP2 level. NEDD4 interacted with the C‐terminal region and ubiquitinated the N‐terminal lysine 88 in PIP5Kα. In addition, PIP5Kα gene disruption inhibited epidermal growth factor (EGF)‐induced Akt activation and caused significant proliferation defect in breast cancer cells. Notably, PIP5Kα K88R mutant that was resistant to NEDD4‐mediated ubiquitination and degradation showed more potentiating effects on Akt activation by EGF and cell proliferation than wild‐type PIP5Kα. Collectively, these results suggest that PIP5Kα is a novel degradative substrate of NEDD4 and that the PIP5Kα‐dependent PIP2 pool contributing to breast cancer cell proliferation through PI3K/Akt activation is negatively controlled by NEDD4.

## 1 INTRODUCTION

Phosphoinositides, the phosphorylated derivatives of phosphatidylinositol, are lipid constituents on cellular membranes. The metabolic pathways and functional roles of phosphatidylinositol 4,5‐bisphosphate (PIP2), one of the phosphoinositides, have been extensively studied because of its significant impacts on membrane signalling, trafficking and dynamics.^1^, ^2^ PIP2 metabolism is also implicated in various human diseases including cancer.^3^, ^4^ Phosphatidylinositol 3‐kinase (PI3K)‐catalysed phosphatidylinositol 3,4,5‐trisphosphate (PIP3) formation from PIP2 is essential for Akt phosphorylation and activation, promoting cancer cell growth and proliferation.^5^ The type I phosphatidylinositol 4‐phosphate 5‐kinase (PIP5K) family members comprising PIP5Kα, PIP5Kβ and PIP5Kγ primarily account for PIP2 production at the plasma membrane by catalysing the phosphorylation of phosphatidylinositol 4‐phosphate.^1^, ^3^

Accumulating evidence has indicated involvement of PIP5K‐dependent PIP2 production in cancer progression. For example, the focal adhesion‐specific PIP5Kγ90 (a 90 kD splice variant of PIP5Kγ) enhances migration, invasion and proliferation of breast cancer cells.^6^, ^7^, ^8^ In addition, PIP5Kα is required for invadopodia formation and promotes survival of breast cancer cells.^9^, ^10^ A previous report also showed that PIP5Kα inhibition using a small compound reduced invasion and caused apoptotic cell death in prostate cancer cells.^11^ It is proposed that the oncogenic property of PIP5Kα is closely linked to the activation of PI3K/Akt signalling pathway.^10^, ^11^

There have been many studies elucidating the underlying mechanisms, by which PIP5K activation is spatiotemporally regulated.^12^, ^13^, ^14^ For instance, PIP5K catalytic activities are modulated by upstream regulators such as Rho family small GTPases, binding partner proteins and post‐translational modifications such as phosphorylation.^12^, ^13^, ^14^, ^15^ However, relatively little is known about the potential regulation of PIP5K via protein stability. To our knowledge, there is actually no evidence of protein degradation of PIP5Kα. In this regard, we have been intrigued by the possibility that PIP5Kα is a target of the ubiquitin‐proteasome system (UPS), one of main degradative pathways, for its regulation, which may also play a role in cancer development.

Among a number of E3 ubiquitin ligases, we especially paid attention to neural precursor cell expressed, developmentally down‐regulated gene 4 (NEDD4) as a candidate for PIP5Kα ubiquitination in that it regulates a wide range of membrane proteins including receptors, ion channels, adaptors and signalling players.^16^, ^17^ NEDD4 (also called NEDD4‐1) is a prototype of the NEDD4 family of E3 ubiquitin ligases and is known to regulate various proteins that are related to cancer.^18^, ^19^ NEDD4 is composed of an N‐terminal C2 domain that binds to calcium or acidic membrane lipids such as phosphoinositides, four central WW domains that recognize the PY motifs (PPXY or LPXY) in target substrates through direct interaction and a C‐terminal catalytic HECT (homologous to the E6‐AP carboxyl terminus) domain that mediates ubiquitination.^17^, ^20^

In this study, we attempted to determine whether NEDD4 could regulate PIP5Kα protein stability. Here, we showed for the first time that NEDD4 interacted with PIP5Kα and mediated its degradation via the UPS, resulting in reduction of PIP2 levels. Our results from PIP5Kα‐deficient and ‐reconstituted breast cancer cells supported that PIP5Kα positively mediates breast cancer cell proliferation and epidermal growth factor (EGF)‐induced Akt activation. Furthermore, a PIP5Kα mutant (K88R) that was resistant to degradation by NEDD4 had greater effects on them compared to wild‐type (WT) PIP5Kα. Overall, these results suggest that proteasomal degradation of PIP5Kα by NEDD4 can be an alternative way for controlling the protein and PIP2 levels leading to suppression of PI3K/Akt‐associated cancer progression.

## 2 MATERIALS AND METHODS

### 2.1 Reagents and antibodies

Most chemicals, including MG132, cycloheximide, DMEM, EGF, anti‐FLAG M2 affinity gels, and antibodies to α‐tubulin, FLAG and PIP5Kγ90, were purchased from Sigma‐Aldrich (St. Louis, MO, USA). Lipofectamine 2000, Lipofectamine RNAiMAX, Opti‐MEM I and V5 antibody were purchased from Thermo Fisher Scientific (Waltham, MA, USA). ISA‐2011B was obtained from MedChem Express (Monmouth, NJ, USA). Antibodies to PIP5Kα, β‐actin and green fluorescent protein (GFP) were from Santa Cruz Biotechnology (Santa Cruz, CA, USA); NEDD4 (Abcam, Cambridge, MA, USA), ubiquitin (Dako, Carpinteria, CA, USA), HA (Covance, Princeton, NJ, USA), phospho‐Akt (S473) and Akt (Cell Signalling Technology, Danvers, MA, USA) and GST (GE Healthcare, Princeton, NJ, USA) were commercially purchased.

### 2.2 Expression constructs

FLAG‐PIP5Kα, Tubby‐R332H‐YFP (yellow fluorescent protein) and FLAG‐PIP5Kγ90 plasmids were previously described.^21^, ^22^ The K88R mutation and the mutation of LPGY to AAGF (193‐196 aa) were introduced into mouse FLAG‐PIP5Kα using a QuikChange II Site‐Directed Mutagenesis Kit (Agilent Technologies, Santa Clara, CA, USA). The WT and/or K88R PIP5Kα inserts were subcloned into the *BamH*I site of pEGFP‐C1 and pGEX‐4T‐3 vectors. The N‐terminal (1‐65 aa), catalytic (66‐434 aa), C‐terminal (435‐546 aa) and the C‐terminally deleted (1‐434 aa) regions of PIP5Kα were amplified by PCR from Myc‐PIP5Kα 22 and subcloned into the *BamH*I‐*Xho*I sites of a pGEX‐6P‐1 and/or a pcDNA3‐FLAG vectors. The mutated and recombinant plasmids were confirmed by DNA sequencing (Genotech, Daejeon, Korea). The HA‐tagged plasmids of ubiquitin (17608), ubiquitin K48R (17604), NEDD4 (27002), NEDD4‐C867A (26999) and mCherry‐NEDD4 (38316) were purchased from Addgene. GFP‐PLCδ‐PH (pleckstrin homology) was provided by Pietro De Camilli (Yale University, New Haven, CT, USA). V5‐tagged NEDD4, NEDD4L and ITCH were provided by Ho Chul Kang (Ajou University, Suwon, Korea).

### 2.3 Cell cultures and transfection

HEK293, HeLa, NCI‐N87, MDA‐MB‐231 and SKBR3 cell lines were cultured in DMEM supplemented with 10% FBS and penicillin/streptomycin (HyClone, Logan, UT, USA) at 37°C in a humidified atmosphere of 5% CO_2_ and 95% air and were routinely subcultured at 2‐ or 3‐day intervals. MCF10A cells were cultured in a 1:1 mixture of DMEM and Ham's F12 media supplemented with 5% horse serum, 20 ng/mL EGF, 0.5 μg/mL hydrocortisone, 100 ng/mL cholera toxin and 10 μg/mL insulin. Cells were cotransfected with the indicated plasmids or corresponding empty vectors with Lipofectamine 2000 for 1 day.^22^ For NEDD4 gene knockdown, double‐stranded siRNAs targeting human NEDD4 (siRNA #1: UAGAGCCUGGCUGGGUUGUUU; siRNA #2: UUCCAUGAAUCUAGAAGAACA) from Bioneer (Daejeon, Korea) were mixed with Lipofectamine RNAiMAX in Opti‐MEM I and added to cells for 2 days. An siRNA (AUUGUAUGCGAUCGCAGACUU) was used as a negative control.

### 2.4 Western blotting and immunoprecipitation

Cell lysate preparation, protein quantification, SDS‐PAGE, Western blotting and anti‐FLAG immunoprecipitation (IP) were performed as described previously.^21^, ^22^ For endogenous PIP5Kα or NEDD4 IP, cell lysates (~1.5 mg) were incubated with 5 μg of the primary antibody for 4 hours at 4°C and the immune complexes were captured with 25 μL of protein G agarose beads (Merck Millipore, Billerica, MA, USA) for an additional 2 hours. To detect PIP5Kα ubiquitination, cells were lysed in a buffer containing *N*‐ethylmaleimide (10 mmol/L) and 10% SDS. After heat treatment (95°C, 5 minutes) and a dilution (1:10), the resulting lysates were processed for the FLAG IP.

### 2.5 GST‐fusion protein pull‐down assay

Glutathione *S*‐transferase (GST) fusion proteins of PIP5Kα were expressed in the *Escherichia coli* strain BL21 and affinity purified using glutathione‐Sepharose 4B beads (GE Healthcare).^21^ After mixing cell lysates (~1.0 mg) for 4 hours at 4°C, the resulting beads were washed with PBS containing 0.1% Tween 20 and analysed by SDS‐PAGE and immunoblotting.

### 2.6 Cell imaging and immunostaining

Fluorescent images were captured with a Zeiss LSM 710 confocal microscope (Carl Zeiss GmbH, Jena, Germany) as previously described.^21^, ^22^ In brief, cells were fixed with 4% paraformaldehyde for 20 minutes. At indicated, cells were immunostained with mouse monoclonal anti‐HA or anti‐FLAG antibody, followed by staining with Alexa Fluor 594‐conjugated secondary antibodies.

### 2.7 PIP5Kα knockout

Cas9‐mediated gene editing was performed by the lentiviral infection of a single guide RNA (sgRNA) and CRISPR/Cas9 system using the lentiCRISPRv2 vector (a gift of Prof. Daesik Lim, KAIST, Daejeon, Korea).^23^, ^24^ The guide RNA sequences used for this study were as follows (Bioneer): upper, 5′‐caccgCGCCCTGCCGGGCTTACCTG‐3′, and bottom, 5′‐aaacCAGGTAAGCCCGGCAGGGCGc‐3′ for human PIP5Kα; upper, caccgATCGTTTCCGCTTAACGGCG, and bottom, 5′‐aaacCGCCGTTAAGCGGAAACGATc‐3′ for a non‐target control. The oligo annealing and subcloning, the lentiviral production and the cell transduction were carried out according to the instructions. Cells were infected with recombinant lentiviruses for 2 days and then cultured with fresh complete media containing puromycin (3.0 μg/mL) for 2 weeks. Puromycin‐resistant clones were isolated and screened for the PIP5Kα gene knockout using Western blot analysis and genomic DNA sequencing.

### 2.8 Colony formation assay

PIP5Kα sgRNA‐ or non‐targeting sgRNA‐expressing cells were seeded in 6‐well plates at a density of 500‐1000 cells/well. For PIP5Kα complementation experiments, FLAG‐PIP5Kα plasmids were transiently transfected into PIP5Kα knockout cells using Lipofectamine 2000 before cell seeding. After 7‐10 days, cells were fixed in an acetic acid:methanol mixture (1:7, v/v) for 1 minutes at room temperature and stained with 0.5% crystal violet, and then the number of cell colonies was counted.

### 2.9 Quantitative real‐time RT‐PCR (qRT‐PCR)

cDNA was synthesized and qRT‐PCR analysis was performed as described previously.^22^, ^25^ The specific primers (Table S1) for E2F transcription factor 1 (E2F1), cyclin‐dependent kinase 1 (CDK1), cyclin D1 gene (CCND1), forkhead box O3 (FOXO3), PIP5Kα and GAPDH (a housekeeping gene) from Bioneer were used. All PCR samples were prepared in triplicate and the relative mRNA expression levels were determined by the 2^−ΔΔCt^ method.

### 2.10 Statistical analysis

All experiments were performed independently at least three times with similar results. Band intensities of Western blots were measured using NIH ImageJ software (National Institutes of Health, Bethesda, MD, USA). Data shown in the graphs are presented as the mean ± SEM. The statistical significance of the data was determined using a one‐way analysis of variance with Tukey's multiple comparison tests using GraphPad Prism software (La Jolla, CA, USA).

## 3 RESULTS

### 3.1 NEDD4 induces the proteasomal degradation of PIP5Kα

**Figure 1:**
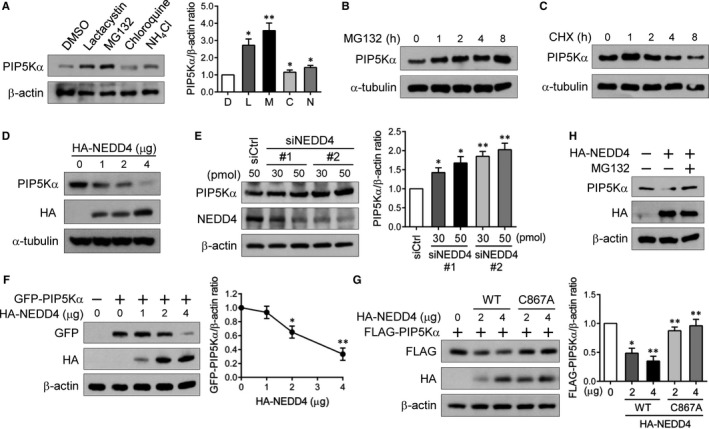
Proteasomal degradation of PIP5Kα by NEDD4. HEK293 cells were treated with lactacystin (L), MG132 (M), chloroquine (C) (each 10 μmol/L), NH_4_Cl (N, 1 mmol/L), or DMSO (D, a vehicle control) for 4 h (A), or with 10 μmol/L MG132 (B) or 10 μmol/L cycloheximide (CHX) (C) for the indicated times. (A‐C) The PIP5Kα protein in cell lysates was analysed by immunoblotting. HEK293 cells were transfected with different amounts of HA‐NEDD4 (D) or with control siRNA or two NEDD4 siRNAs (E), as indicated. HEK293 cells were cotransfected with GFP‐PIP5Kα and HA‐NEDD4 (F) or with the WT or inactive (C867A) HA‐NEDD4 together with FLAG‐PIP5Kα (G). (H) HA‐NEDD4‐transfected cells were left untreated or treated with MG132 (10 μmol/L) for 1 h. (D‐H) Cell lysates were analysed by Western blotting with the indicated antibodies. As a loading control, β‐actin or α‐tubulin was included. (A, E‐G) PIP5Kα levels were normalized to β‐actin levels and quantified relative to those in cells with untreated control (A), control siRNA (E) or without HA‐NEDD4 (F, G). Values in the graphs are presented as the mean ± SEM. *P < .05, **P < .01

As a first step to examine the protein stability of PIP5Kα, we tested the possibility of its proteasomal or lysosomal degradation. Changes in PIP5Kα protein levels were analysed by immunoblotting 4 hours after the treatment of HEK293 cells with proteasome inhibitors (lactacystin and MG132), lysosome inhibitors (chloroquine and NH_4_Cl) or DMSO as a vehicle control. PIP5Kα protein levels were significantly enhanced following lactacystin and MG132 treatment but were relatively less affected by chloroquine and NH_4_Cl treatment (Figure 1A). PIP5Kα protein levels continuously accumulated for up to 8 hours during the time course of MG132 treatment (Figure 1B). Conversely, treatment with cycloheximide, an inhibitor of protein synthesis, gradually decreased PIP5Kα protein levels in the same time ranges (Figure 1C).

The HECT domain‐containing E3 ubiquitin ligase NEDD4 plays important roles in regulating various proteins present in the plasma membrane.^16^, ^17^ Here, we examined NEDD4 for its potential effect on PIP5Kα protein levels. Transfected HA‐tagged NEDD4 induced degradation of endogenous PIP5Kα in a dose‐dependent manner (Figure 1D). In contrast, when other NEDD4 family members NEDD4L and ITCH 20 were similarly tested, NEDD4L and ITCH did not show a degrading effect on PIP5Kα (Figure S1). As expected, PIP5Kα mRNA levels were not significantly changed under the same conditions (Figure S2), indicating that the decreased PIP5Kα protein levels by NEDD4 are a post‐translational event. NEDD4 gene knockdown with two different siRNAs resulted in relatively high levels of PIP5Kα compared to the control siRNA (Figure 1E). We also examined the effects of NEDD4 on transiently expressed PIP5Kα with cells cotransfected with HA‐NEDD4 and GFP‐tagged PIP5Kα. The dose‐dependently expressed HA‐NEDD4 reciprocally altered GFP‐PIP5Kα levels (Figure 1F). The catalytically inactive C867A mutant of HA‐NEDD4 was less effective in degrading FLAG‐PIP5Kα compared to the WT NEDD4, as expected (Figure 1G). In addition, pre‐treatment with MG132 blocked the degradation of endogenous PIP5Kα by HA‐NEDD4 (Figure 1H). All these results indicate that NEDD4 induces the proteasomal degradation of PIP5Kα. However, endogenous and transfected PIP5Kγ90 was not degraded by NEDD4 (Figure S3), supporting the specificity of NEDD4‐mediated PIP5Kα degradation.

### 3.2 NEDD4 mediates ubiquitination of PIP5Kα

**Figure 2:**
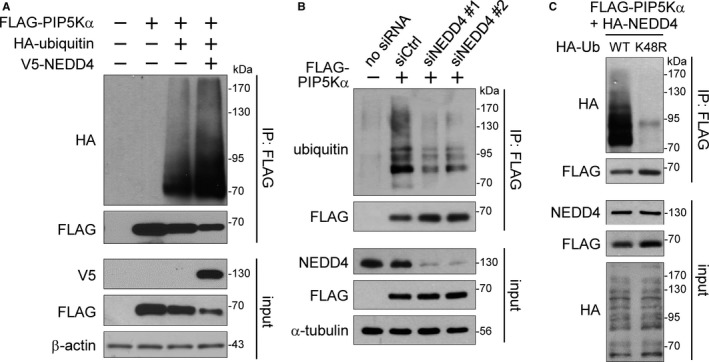
NEDD4‐mediated ubiquitination of PIP5Kα. FLAG‐PIP5Kα was cotransfected into HEK293 cells together with HA‐ubiquitin and/or V5‐NEDD4 (A) or with HA‐NEDD4 and WT or K48R HA‐ubiquitin (Ub) (C). (B) HEK293 cells were treated with control siRNA or NEDD4 siRNAs and were then transfected with FLAG‐PIP5Kα. (A‐C) Cell lysates (input) were subjected to FLAG IP, and the input and FLAG IP samples were analysed by Western blotting with the indicated antibodies

We examined whether PIP5Kα is ubiquitinated by NEDD4. For this, FLAG‐PIP5Kα was affinity purified by anti‐FLAG immunoprecipitation (IP) from HEK293 cells expressing FLAG‐PIP5Kα, HA‐ubiquitin and/or V5‐NEDD4. Cell lysates were completely denatured prior to the IP experiments to avoid possible ubiquitination of PIP5Kα‐associated proteins as described in Materials and Methods. HA immunoblotting of the FLAG immunoprecipitates revealed the multiple, higher molecular weight bands greater than 70 kD (non‐modified FLAG‐PIP5Kα) in the presence of FLAG‐PIP5Kα, HA‐ubiquitin and V5‐NEDD4 (Figure 2A). The transient knockdown of NEDD4 by siRNAs increased the levels of immunoprecipitated FLAG‐PIP5Kα but decreased the ubiquitinated levels of FLAG‐PIP5Kα compared to the control siRNA knockdown (Figure 2B). These observations support that PIP5Kα is a novel NEDD4 substrate for protein ubiquitination. Protein substrates to be degraded by the UPS are polyubiquitinated through K48‐linked ubiquitin chains.^26^ We tested the possibility that NEDD4 can mediate K48‐linked ubiquitin chains on PIP5Kα by taking advantage of a mutant (K48R) ubiquitin that cannot form the K48‐linked chains. The polyubiquitination levels of PIP5Kα were significantly reduced in the ubiquitin K48R‐expressing cells compared to the WT ubiquitin‐expressing cells (Figure 2C). The FLAG‐PIP5Kα protein levels in the input and FLAG immunoprecipitates were higher in the ubiquitin K48R‐expressing cells than in the WT ubiquitin‐expressing cells (Figure 2C). These results indicate that PIP5Kα degradation by NEDD4 is mediated via K48‐linked ubiquitination.

### 3.3 PIP5Kα interacts with NEDD4 through its C‐terminal region

**Figure 3:**
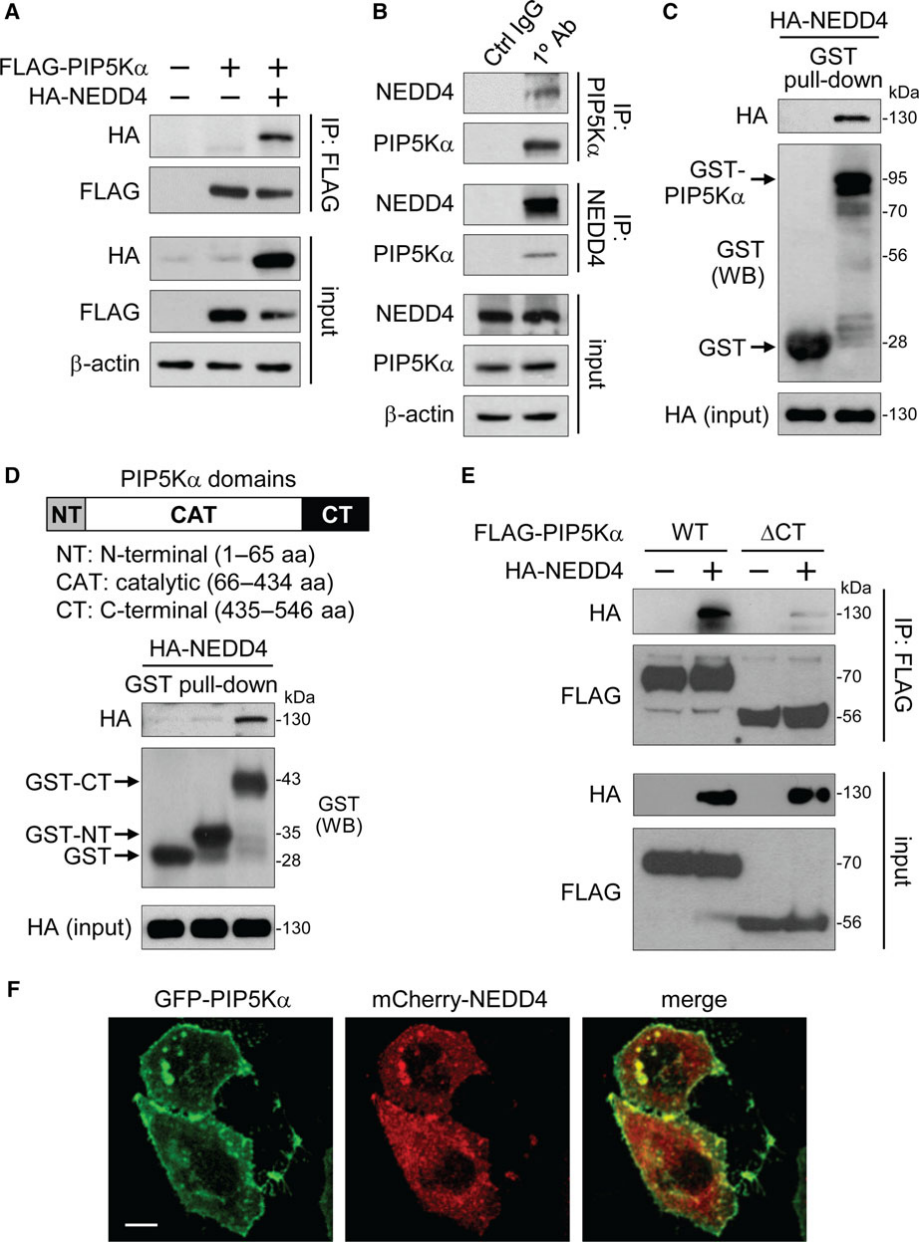
Binding of the PIP5Kα C‐terminal region to NEDD4. (A) FLAG IP products were prepared from HEK293 cells transfected with FLAG‐PIP5Kα and/or HA‐NEDD4. (B) HEK293 cell lysates were immunoprecipitated with the anti‐PIP5Kα antibody, anti‐NEDD4 antibody or their corresponding control IgG. (A, B) Input and resulting immunoprecipitates were immunoblotted with the indicated antibodies. GST‐fusion proteins of the full‐length PIP5Kα (C), the N‐terminal or C‐terminal region of PIP5Kα (D) or the GST control protein conjugated to glutathione beads were mixed with HEK293 cell lysates expressing HA‐NEDD4. (C, D) The bound and input HA‐NEDD4 and comparable levels of GST‐fusion proteins, indicated by the arrows, were revealed by HA and GST immunoblottings, respectively. (E) Cells transfected with FLAG‐PIP5Kα WT or ∆CT in the absence and presence of HA‐NEDD4 were processed in the same way as (A). (F) Confocal images of cotransfected GFP‐PIP5Kα and mCherry‐NEDD4. Scale bar, 10 μm

The ubiquitination of target proteins by NEDD4 requires their physical interaction with NEDD4. We tested a possible interaction between NEDD4 and PIP5Kα with cells transfected with FLAG‐PIP5Kα and/or HA‐NEDD4. HA‐NEDD4 coprecipitated with FLAG‐PIP5Kα only when both proteins were coexpressed (Figure 3A). We carried out PIP5Kα or NEDD4 IP in intact HEK293 cells. Anti‐NEDD4 immunoblotting of PIP5Kα immunoprecipitates showed that endogenous NEDD4 coprecipitated with PIP5Kα, and likewise, the coprecipitation of PIP5Kα and NEDD4 was observed in NEDD4 IP samples (Figure 3B). No immunoreactivity with PIP5Kα and NEDD4 in IP samples with control IgG confirmed the specificity of this experiment. Alternatively, we generated a GST‐fusion protein of full‐length PIP5Kα. A control GST and GST‐PIP5Kα protein conjugated to glutathione‐Sepharose beads were used in the pull‐down assay with HA‐NEDD4‐expressing cell lysates. HA immunoblotting showed that HA‐NEDD4 was pulled down by GST‐PIP5Kα but not by GST alone (Figure 3C).

The PPXY or LPXY motifs in NEDD4 substrates mediate the NEDD4 binding^27^ and PIP5Kα contains a single LPGY motif (193‐196 aa) in the catalytic domain (Figure S4A). To examine whether this LPGY motif is responsible for NEDD4 binding, we introduced mutations (LPGY→AAGF) into the FLAG‐PIP5Kα. Somewhat unexpectedly, however, the AAGF mutant retained almost the same binding activity as the WT for HA‐NEDD4 (Figure S4B) and was still degradable by NEDD4 at levels comparable to those of the WT (Figure S4B). Hence, as an alternative approach, we made GST‐fusion proteins of the N‐terminal (1‐65 aa) and C‐terminal (435‐546 aa) regions of PIP5Kα. The central catalytic domain (66‐434 aa) was omitted simply because of poor solubility and low yield. HA immunoblotting revealed the presence of HA‐NEDD4 only in the GST‐C‐terminal pull‐down sample (Figure 3D). HA‐NEDD4 also coprecipitated with FLAG‐tagged PIP5Kα C‐terminal region (Figure S4C). Consistently, the C‐terminal deletion mutant of PIP5Kα (PIP5Kα ∆CT) lost the binding affinity for NEDD4 (Figure 3E). These results indicate that the C‐terminal region of PIP5Kα, but not its LPGY motif, interacts with NEDD4. The FLAG‐PIP5Kα C‐terminal region interfered with the degrading effect of HA‐NEDD4 on endogenous PIP5Kα (Figure S4D), supporting the specific binding of the PIP5Kα C‐terminal region to NEDD4.

Based on these results, we tested the colocalization between these two proteins. GFP‐PIP5Kα seemed to localize to the plasma membrane and form membranous puncta and aggregates, and mCherry‐NEDD4 was also, at least partially, found on the same membrane sites although it also showed a cytoplasmic distribution (Figure 3F).

### 3.4 NEDD4 expression leads to a decrease in PIP2 levels

**Figure 4:**
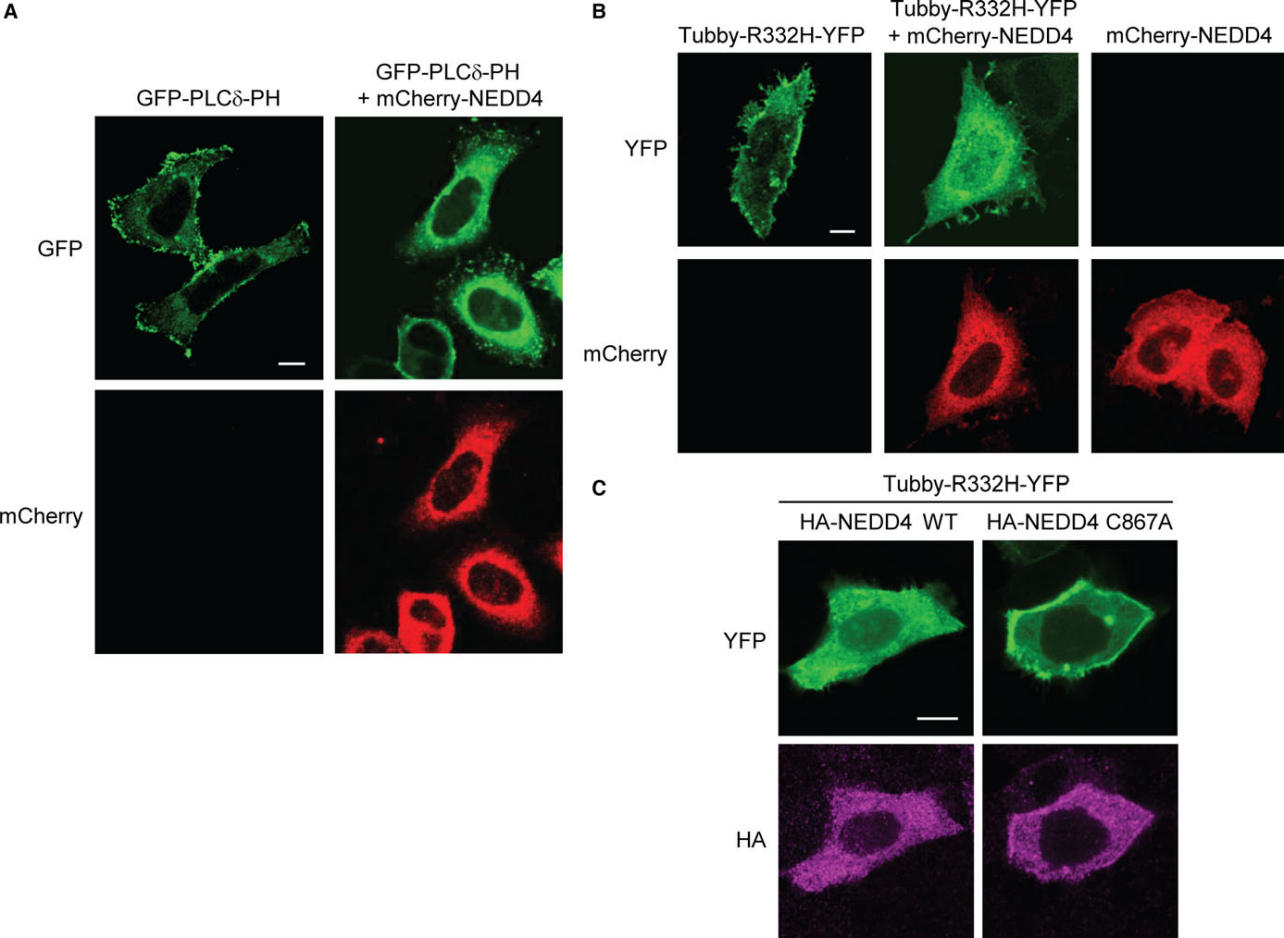
Reduction of the PIP2 level by NEDD4. HeLa cells were cotransfected with GFP‐PLCδ‐PH (A) or Tubby‐R332H‐YFP (B) in the absence or presence of mCherry‐NEDD4, as indicated. Cells were fixed and the fluorescence images of GFP and mCherry (A) or YFP and mCherry (B) were captured using confocal microscopy. (C) HeLa cells were cotransfected with Tubby‐R332H‐YFP and WT or C867A HA‐NEDD4. After fixation, cells were immunostained with the anti‐HA antibody and then with the Alexa Fluor 594‐labelled secondary antibody. HA immunofluorescence in magenta colour and YFP fluorescence were visualized by confocal microscopy. Scale bars, 10 μm

We examined the possibility that the NEDD4‐mediated degradation of PIP5Kα could influence PIP2 levels using the GFP‐tagged PH domain of PLCδ (GFP‐PLCδ‐PH) that has been used as a PIP2 reporter.^28^ The fluorescent protein having a binding affinity for PIP2 undergoes translocation between the plasma membrane and cytoplasmic space depending on the lipid levels. A prominent GFP fluorescence of the PIP2 probe was found at the cell surface when it was solely transfected (Figure 4A). Upon cotransfection with mCherry‐NEDD4, GFP‐PLCδ‐PH diffused into the cytoplasm, indicative of a reduction in PIP2 levels (Figure 4A). Similarly, we used another fluorescent construct of the membrane‐bound transcription factor Tubby for monitoring PIP2 levels. Tubby selectively binds to plasma membrane PIP2 through its Tubby domain, and the mutated (R332H) Tubby more sensitively reflects PIP2 changes.^29^, ^30^ Transfected Tubby‐R332H‐YFP alone localized to the plasma membrane as expected, but it became concentrated in the perinuclear sites by cotransfection with mCherry‐NEDD4 (Figure 4B). We compared the effects of WT and catalytically inactive NEDD4 on PIP2 levels. Tubby‐R332H‐YFP still localized to the cell surface in the cells expressing HA‐NEDD4 C867A, whereas it was relatively soluble in HA‐NEDD4 WT‐expressing cells (Figure 4C). The NEDD4‐induced membrane‐to‐cytosol translocation of Tubby‐R332H‐YFP further supports that PIP5Kα degradation by NEDD4 leads to a decrease in PIP2 levels.

### 3.5 PIP5Kα is ubiquitinated by NEDD4 at the lysine 88 residue

**Figure 5:**
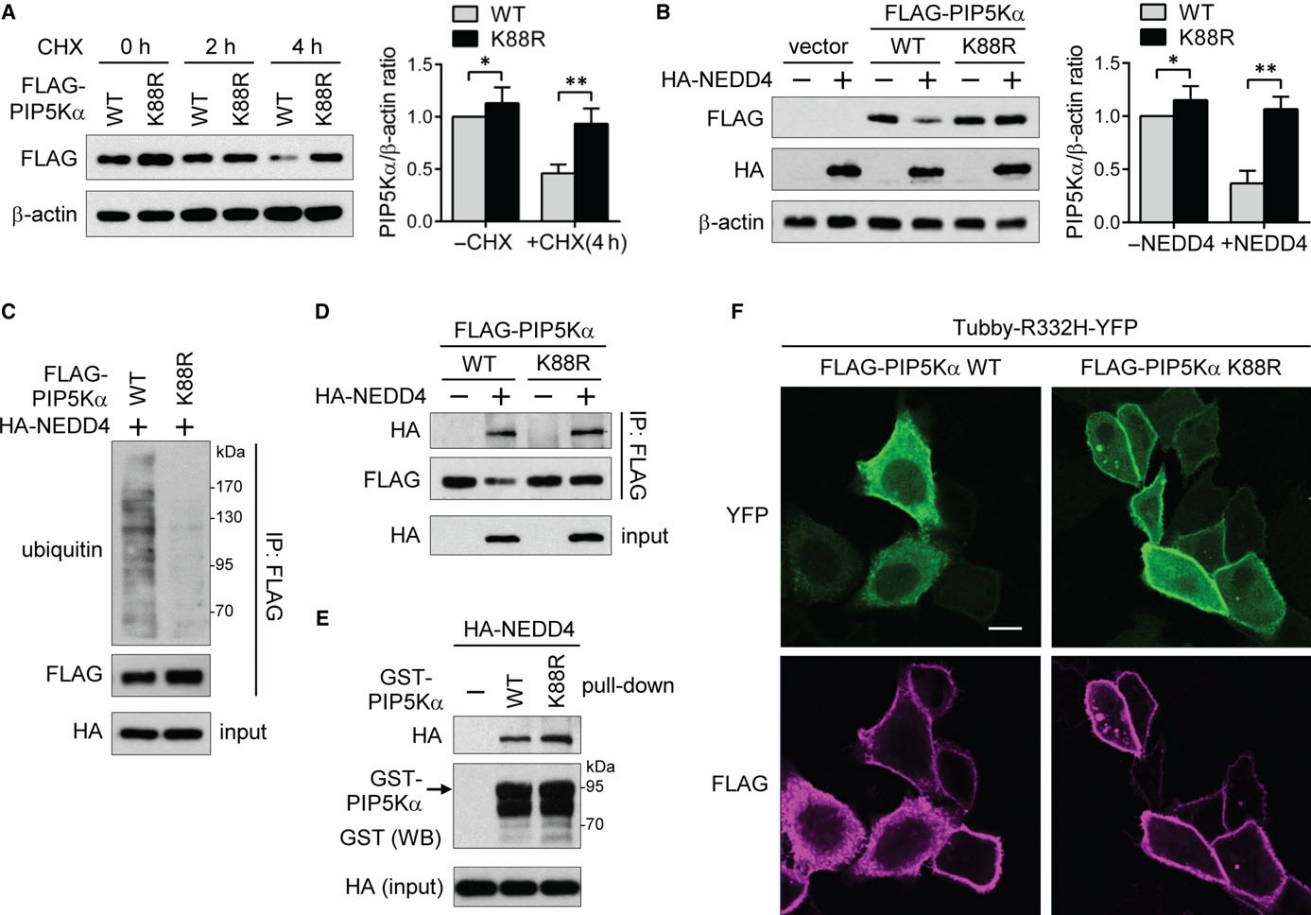
PIP5Kα K88 is the main ubiquitination site for NEDD4. (A) HEK293 cells were treated with or without 10 μmol/L cycloheximide (CHX) for the indicated times at 1 day post‐transfection of WT or K88R FLAG‐PIP5Kα. (B‐D) HEK293 cells were cotransfected with WT or K88R FLAG‐PIP5Kα in the presence or absence of HA‐NEDD4. (A, B) FLAG‐PIP5Kα levels measured by immunoblotting were normalized to β‐actin (a loading control) levels and quantified relative to those in the cells transfected with WT FLAG‐PIP5Kα without CHX (A) or HA‐NEDD4 (B). Values in the graphs are presented as the mean ± SEM. *P < .05, **P < .01. (C, D) FLAG immunoprecipitates and cell lysates (input) were processed for Western blot analysis with the indicated antibodies. (E) Cell lysates expressing HA‐NEDD4 were pulled down with GST‐tagged full‐length WT or K88R PIP5Kα. The bound and input HA‐NEDD4 and GST‐PIP5Kα bait proteins were detected by Western blotting. (F) HeLa cells cotransfected with Tubby‐R332H‐YFP and with either WT or K88R FLAG‐PIP5Kα were immunostained with an anti‐FLAG antibody and followed by staining with the Alexa Fluor 594‐labelled secondary antibody. FLAG immunofluorescence in magenta colour and YFP fluorescence images were captured by confocal microscopy. Scale bar, 10 μm

As an attempt to determine the specific lysine residue mediating PIP5Kα degradation by NEDD4, we utilized several ubiquitination site prediction methods. The lysine in the STKPER motif is shown to have a relatively high probability among multiple candidates, and the equivalent lysine 97 of human PIP5Kγ90 was reported to be ubiquitinated by HECTD1, a HECT domain‐containing E3 ubiquitin ligase.^7^ Thus, we substituted K88, the corresponding residue in mouse PIP5Kα, with arginine and assessed the protein stability of WT and K88R PIP5Kα under cycloheximide treatment conditions. Transfected FLAG‐PIP5Kα WT underwent time‐dependent degradation up to 4 hours after the addition of cycloheximide, but the K88R mutant remained at relatively high levels (Figure 5A). The PIP5Kα K88R mutant remained stable irrespective of the presence of HA‐NEDD4 in contrast to WT PIP5Kα (Figure 5B) and was resistant to NEDD4‐mediated ubiquitination unlike the PIP5Kα WT (Figure 5C).

We compared binding affinities for HA‐NEDD4 between the WT and K88R FLAG‐PIP5Kα. The K88R mutant maintained a binding affinity for NEDD4 as strong as the WT (Figure 5D). Moreover, we repeated the pull‐down assay with full‐length GST‐fusion proteins of PIP5Kα WT and K88R. Consistently, the NEDD4‐binding abilities of the GST‐PIP5Kα K88R were similar to those of the GST‐PIP5Kα WT (Figure 5E), suggesting that the non‐degradable PIP5Kα K88R mutant against NEDD4 is likely due to a defect in ubiquitination rather than in binding. WT or K88R FLAG‐PIP5Kα‐cotransfected cells with Tubby‐R332H‐YFP were examined for PIP2 levels by monitoring YFP localization. Confocal images showed that the plasma membrane targeting of the PIP2‐specific fluorescent probe was more robust in the K88R mutant‐expressing cells than in the WT‐expressing cells (Figure 5F), indicating a relatively high level of PIP2 in the K88R mutant. Collectively, these data support that K88 is one of the main degradative ubiquitination sites of PIP5Kα by NEDD4.

### 3.6 PIP5Kα K88R potentiates Akt activation and breast cancer cell proliferation

**Figure 6:**
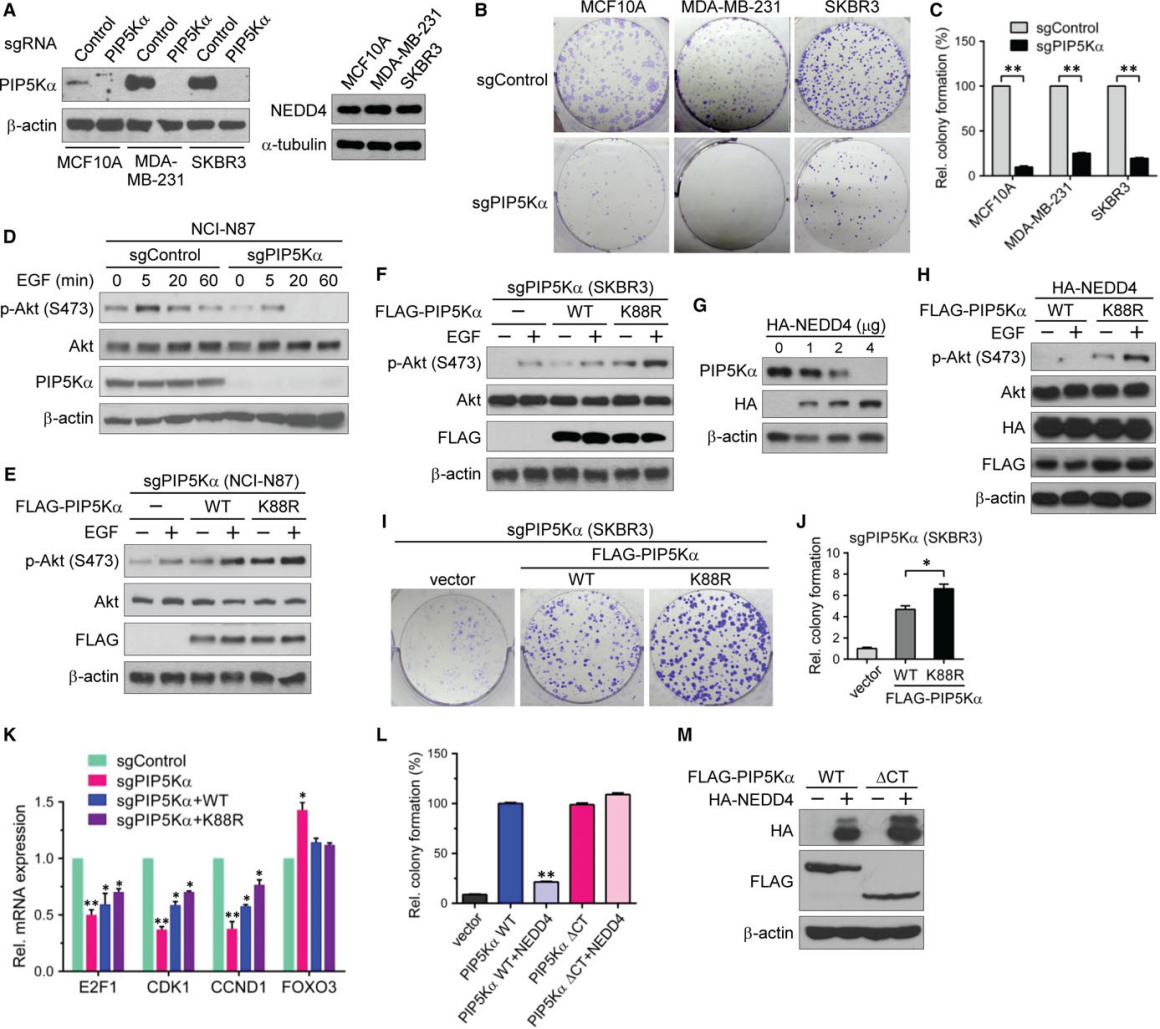
NEDD4‐mediated PIP5Kα degradation is involved in Akt signalling and cancer cell proliferation. (A, B) MCF10A, MDA‐MB‐231 and SKBR3 cells were infected with CRISPR/Cas9‐based lentivirus expressing PIP5Kα‐targeting sgRNA (sgPIP5Kα) or non‐targeting sgRNA (sgControl). (A) Western blot analysis of PIP5Kα and NEDD4. (B, C) Colony formation assay results. (D) sgControl and sgPIP5Kα were introduced into NCI‐N87 cells in the same way as in (A). PIP5Kα knockout (sgPIP5Kα) NCI‐N87 (E) and SKBR3 (F) cells were reconstituted with the empty vector, WT or K88R FLAG‐PIP5Kα. (D‐F) After overnight serum starvation, cells were treated with or without EGF (100 ng/mL) for the indicated times (D) or 5 min (E, F). Phosphorylated (S473) and total Akt and endogenous or transfected PIP5Kα were examined by Western blotting. (G) Western blot analysis of PIP5Kα in SKBR3 cells transfected with the indicated amounts of HA‐NEDD4. (H) PIP5Kα knockout SKBR3 cells were cotransfected with HA‐NEDD4 and FLAG‐PIP5Kα WT or K88R, and then treated with or without EGF, as indicated. Resulting cell lysates were examined by immunoblotting with the indicated antibodies. (I, J) Colony formation assay results with the PIP5Kα knockout SKBR3 cells reconstituted with FLAG‐PIP5Kα WT or K88R. (K) Control and PIP5Kα knockout SKBR3 cells and the PIP5Kα WT‐ or K88R‐reconstituted cells were processed for qRT‐PCR analysis of the indicated genes. (L) Colony formation assay results with PIP5Kα knockout SKBR3 cells reconstituted with FLAG‐PIP5Kα WT or ∆CT in the presence or absence of HA‐NEDD4. (M) Cell lysates in (L) were analysed for the transfected proteins by Western blotting. The surviving colonies (C, J, L) and mRNA expression levels (K) were quantified relative to those in the respective sgControl (C, K), vector control (J), or FLAG‐PIP5Kα WT‐reconstituted (L) cells. Values in the graphs are presented as the mean ± SEM. *P < .05, **P < .01

It has been reported that PIP5Kα plays a role in cancer progression.^9^, ^10^, ^11^ In this regard, we examined whether NEDD4‐induced PIP5Kα degradation might be implicated in cancer. To test this idea, we first developed PIP5Kα knockout in breast cancer cell lines (MDA‐MB‐231 and SKBR3) and a normal breast epithelial cell line (MCF10A) using a lentiviral expression system encoding a CRISPR/Cas9‐mediated sgRNA targeting PIP5Kα (sgPIP5Kα) or a non‐targeting sgRNA (sgControl) as a negative control. We confirmed that PIP5Kα protein expression levels in the sgPIP5Kα‐expressing cells were clearly reduced compared to those in the sgControl‐expressing cells (Figure 6A). We observed comparable expression levels of endogenous NEDD4 protein in the three breast cancer cells (Figure 6A). Interestingly, when we performed the colony formation assay, we observed that PIP5Kα depletion by sgPIP5Kα caused a significant decrease in the number of surviving colonies, suggesting that PIP5Kα is required for cell proliferation (Figure 6B and C). Consistent with this result, inhibition of PIP5Kα with a small compound ISA‐2011B 11 led to defective proliferation in SKBR3 cells (Figure S5A).

The activation of the PI3K/Akt signalling pathway plays a central in growth factor‐induced cell growth and proliferation.^31^ We thus examined the effect of PIP5Kα knockout on Akt activation by EGF. For this, we introduced the lentiviral sgControl and sgPIP5Kα expression into a gastric cancer cell line, NCI‐N87, as described in Figure 6A and then measured EGF‐induced Akt phosphorylation at the S473 residue, a marker of PI3K/Akt activation. EGF stimulation rapidly increased phospho‐Akt to the maximal level within 5 min, followed by dephosphorylation to the basal level during 60 min in sgControl cells (Figure 6D). In contrast, Akt phosphorylation was blunted in PIP5Kα knockout cells during the time course of EGF stimulation (Figure 6D). PIP5Kα knockout also decreased colony formation in NCI‐N87 cells (Figure S5B). The PIP5Kα knockout NCI‐N87 cells were reconstituted with WT or K88R FLAG‐PIP5Kα by transient transfection. We found that reconstitution with the PIP5Kα K88R mutant could more significantly potentiate Akt phosphorylation by EGF than WT PIP5Kα reconstitution (Figure 6E). When we tested the same PIP5Kα reconstitution experiments with PIP5Kα knockout SKBR3 cells, similar results were obtained (Figure 6F). As expected, the NEDD4‐mediated PIP5Kα degradation was detectable in SKBR3 cells as evidenced by immunoblot analysis (Figure 6G), which was consistent with the result in Figure 1D. Furthermore, when HA‐NEDD4 was coexpressed in PIP5Kα‐reconstituted SKBR3 cells, EGF‐induced Akt phosphorylation was also much higher in the K88R‐reconstituted cells than in the WT‐reconstituted cells (Figure 6H).

**Figure 7:**
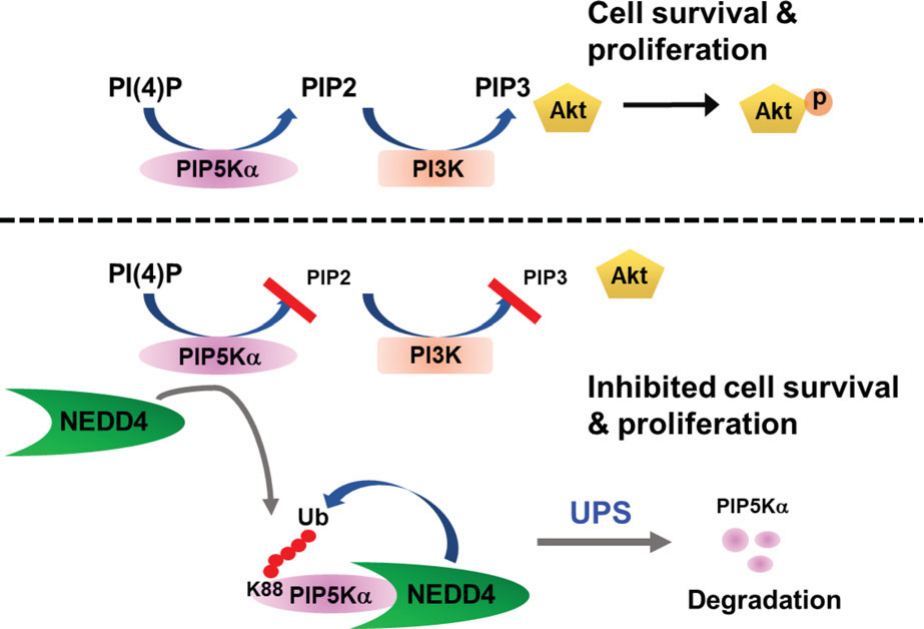
A model for regulation of cancer cell proliferation by PIP5Kα that is a target of NEDD4‐dependent proteasomal degradation. PIP5Kα that produces PIP2 from phosphatidylinositol 4‐phosphate (PI(4)P) contributes to cancer cell survival and proliferation by supplying PIP2, a substrate of PI3K for PIP3 formation, which activates its downstream Akt (top). The PIP2 levels are reduced by NEDD4‐mediated PIP5Kα degradation via UPS, leading to downregulation of the oncogenic PI3K/Akt signalling pathway (bottom)

We tested the potential effects of reconstituted WT and K88R FLAG‐PIP5Kα on cell proliferation by performing the colony formation assay with PIP5Kα knockout SKBR3 cells. Defects in colony formation by PIP5Kα knockout were recovered by the WT, and the K88R mutant had a greater colony‐forming ability (Figure 6I and J). We assessed transcriptional changes in E2F1, CDK1 and CCND1 whose expression levels correlate with increased cell proliferation and are, in general, elevated in various cancers including breast cancer cells.^32^, ^33^, ^34^, ^35^ As shown in Figure 6K, the mRNA expression levels of E2F1, CDK1 and CCND1 were reduced, but conversely, the mRNA levels of the tumour suppressor FOXO3 36, 37 were increased in the PIP5Kα knockout SKBR3 cells compared to those in non‐targeting control cells. Moreover, the transient re‐expression of WT PIP5Kα in the PIP5Kα knockout cells at least partially reversed those changes, and similar rescuing effects of the PIP5Kα K88R mutant on the expression of E2F1, CDK1 and CCND1 were relatively high compared to the WT to some extent (Figure 6K). Reconstitution of the NEDD4 binding‐deficient PIP5Kα ∆CT that harbours the catalytic domain (Figure 3D and E) also increased colony formation as much as the WT (Figure 6L). Notably, HA‐NEDD4 coexpression suppressed colony formation by PIP5Kα WT, but, in contrast, did not show such inhibitory effect on the PIP5Kα ∆CT (Figure 6L). The relevant expressions of transfected HA‐NEDD4 and FLAG‐PIP5Kα proteins were confirmed by Western blot analysis (Figure 6M). Taken together, all these results suggest that PIP5Kα acts upstream of PI3K/Akt signalling by supplying the PI3K substrate PIP2, leading to increased cell proliferation and that NEDD4 serves as a negative regulator of this signalling by inducing proteasomal degradation of PIP5Kα (Figure 7).

## 4 DISCUSSION

In this present study, we showed that PIP5Kα is a ubiquitinated protein that undergoes proteasomal degradation under steady‐state conditions, suggesting the dynamic control of PIP5Kα protein levels through the ubiquitin‐proteasome pathway. Our data revealed that NEDD4 is a reliable candidate to mediate the UPS‐mediated proteolysis of PIP5Kα. As a result, NEDD4 could reduce PIP5Kα‐dependent plasma membrane PIP2 pool. We also demonstrated that K88 of PIP5Kα is the likely residue ubiquitinated by NEDD4. Accordingly, the PIP5Kα K88R mutant enhanced the protein stability of PIP5Kα. The mutants of PIP5Kα K88R, as well as the catalytically inactive NEDD4, led to relatively high levels of plasma membrane PIP2 compared to their WT controls. Considering the fact that many studies of PIP5Ks have focused on the identification of the underlying regulatory mechanisms of their catalytic activities,^12^, ^13^, ^14^, ^15^ this study presents another perspective on the regulation of PIP5K‐dependent PIP2 generation through the NEDD4‐dependent control of PIP5K protein stability.

Our results indicated that NEDD4 interacts with the C‐terminal region of PIP5Kα that does not harbour the NEDD4‐binding P(L)PXY motifs. Similarly, a previous study showed that a non‐P(L)PXY motif of activated fibroblast growth factor receptor 1 was responsible for NEDD4 binding in the receptor's ubiquitination and endocytosis by NEDD4.^38^ In addition, the interaction of PIP5Kγi5, a splice variant of PIP5Kγ, with NEDD4 was mediated through its C‐terminal region that contains no P(L)PXY motifs, and this interaction suppressed NEDD4‐mediated ubiquitination and proteasomal degradation of the tumour suppressor, mitogen‐inducible gene 6, without PIP5Kγi5 degradation.^39^ Apparently, NEDD4 did not mediate PIP5Kγ90 degradation, suggesting a differential regulation of PIP5Kα and PIP5Kγ90 by NEDD4.

Our results showed that PIP5Kα is necessary for breast cancer cell proliferation, as evidenced by the increases in the cell proliferation markers E2F1, CDK1 and CCND1 and the decrease in the tumour suppressor FOXO3. The PIP5Kα K88R mutant that was not degraded by NEDD4 produced greater amounts of PIP2 probably due to its enhanced protein stability and was more potent in promoting activation of the PI3K/Akt signalling pathway by EGF and colony formation than the WT PIP5Kα. Furthermore, the PIP5Kα K88R mutant showed relatively stronger effects on E2F1, CDK1 and CCND1 transcription than the WT PIP5Kα. It is well known that the aberrant activation of PI3K/Akt pathway is one of the primary causes of diverse cancers.^5^ The activation of PI3K/Akt signalling through PIP5Kα has been identified in breast cancer and prostate cancer models.^10^, ^11^ Therefore, a significant implication of this study is that the regulation of PIP5Kα by NEDD4 can play a role in limiting activation of the PI3K/Akt pathway linked to breast cancer progression (Figure 7).

Previous studies showed that mRNA and protein expression levels of PIP5Kα were highly elevated in breast cancer cells 9 and prostate cancer cells,^11^ respectively. Similarly, a previous study using human breast cancer database revealed that only PIP5Kα showed increased gene expression among PIP5K family members.^40^ We also observed more abundant PIP5Kα protein in the breast cancer cells (MDA‐MB‐231 and SKBR3) compared to the normal breast cells (MCF10A) (Figure 6A). These findings imply that PIP5Kα overexpression may be a marker of breast cancer progression. At some point, it may be plausible that the overexpressed PIP5Kα cannot be completely degraded by NEDD4, resulting in accumulation of PIP5Kα protein. Apart from the increased PIP5Kα expression, it may also be possible that a defect in NEDD4‐mediated PIP5Kα degradation causes such high levels of PIP5Kα protein in breast cancer cells. Overall, it will be interesting to investigate those possibilities with efforts to clarify as yet undefined molecular mechanisms.

On the other hand, it has been demonstrated that NEDD4 mediates ubiquitination and degradation of various cancer‐related proteins. For example, NEDD4 degrades the tumour suppressors, such as phosphatase and tensin homologue (PTEN), a lipid phosphatase that hydrolyses PIP3 41 and Beclin 1.^42^ However, NEDD4 is shown to be dispensable for the PTEN regulation.^43^ In addition, the oncoproteins such as Ras 44 and Myc 45 are targeted for degradation by NEDD4. It seems that NEDD4 can distinctly regulate degradative ubiquitination of those different types of protein substrates in various cancer models, which leads to promotion or suppression of tumorigenesis. Based on this study, our results suggest that NEDD4 can act as a suppressor of breast cancer by negatively regulating PIP5Kα.

PIP2 is a critical regulator of a wide range of membrane signalling, vesicle trafficking and actin dynamics, and such pleiotropic roles played by PIP2 are mediated through its binding to and regulatory effect on related cytosolic proteins.^1^, ^2^, ^3^, ^12^, ^13^ NEDD4 also plays an important role in regulating various membrane proteins of receptors, channels and adaptors at the cell surface.^16^, ^17^ In addition, the N‐terminal C2 domain expressed in NEDD4 is known as a recognition module for calcium and phosphoinositides including PIP2,^46^ and Smurf, a NEDD4 family member, was proposed to be activated upon binding of its C2 domain to phosphoinositides.^47^ Our results indicated the physical interaction between NEDD4 and PIP5Kα. These results raise the possibility that NEDD4 may be functionally implicated in the physiological roles of PIP5Kα‐dependent PIP2.

In conclusion, our present results demonstrate that PIP5Kα undergoes proteasomal degradation through interaction with and ubiquitination by the ubiquitin ligase NEDD4, which is accompanied by a reduction in plasma membrane PIP2 levels. Thus, this study proposes an alternative way of controlling PIP2 through NEDD4. Our results indicate that PIP5Kα‐dependent PIP2 production contributes to the activation of PI3K/Akt signalling and breast cancer cell proliferation, which can be impeded by NEDD4, at least partially, through UPS‐mediated PIP5Kα degradation. This study further implicates that PIP5Kα may be a novel anticancer therapeutic target.

## CONFLICT OF INTERESTS

The authors declare no conflict of interests.

## AUTHOR CONTRIBUTIONS

MHT, ES and SYL developed the study concept and design and interpreted the data. MHT, ES, SM, QTN, JC and UJL performed the experiments and analysed and validated the data. SSH, HK, AM and IJ supported the data interpretation. SYL supervised this study and wrote the manuscript.
