# Supplementary material for: NEDD4‐induced degradative ubiquitination of phosphatidylinositol 4‐phosphate 5‐kinase α and its implication in breast cancer cell proliferation
Source: J Cell Mol Med. 2018 May 30;22(9):4117–29. doi: 10.1111/jcmm.13689 (PMC6111810; doi:10.1111/jcmm.13689)
Supplement: Supplementary file 1 [file JCMM-22-4117-s001.docx]

**Supporting information**

**
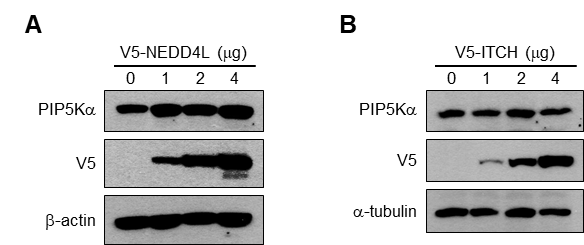
**

**Supplementary Fig. 1** NEDD4L and ITCH do not induce PIP5Kα degradation. HEK293 cells were transfected with different amounts of V5-NEDD4L (**A**) or V5-ITCH (**B**), as indicated. Cell lysates were analyzed for the protein levels of endogenous PIP5Kα and transfected proteins by immunoblotting with the indicated antibodies. As a loading control, β-actin or α-tubulin was included.

**
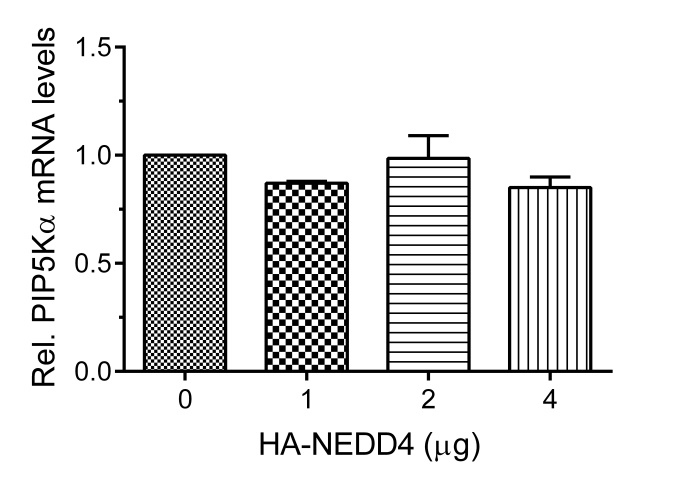
**

**Supplementary Fig. 2** Effect of transfected HA-NEDD4 on PIP5Kα transcriptional level. HEK293 cells were transiently transfected with the indicated amounts of HA-NEDD4 and then mRNA expression levels of PIP5Kα were examined by qRT-PCR analysis.


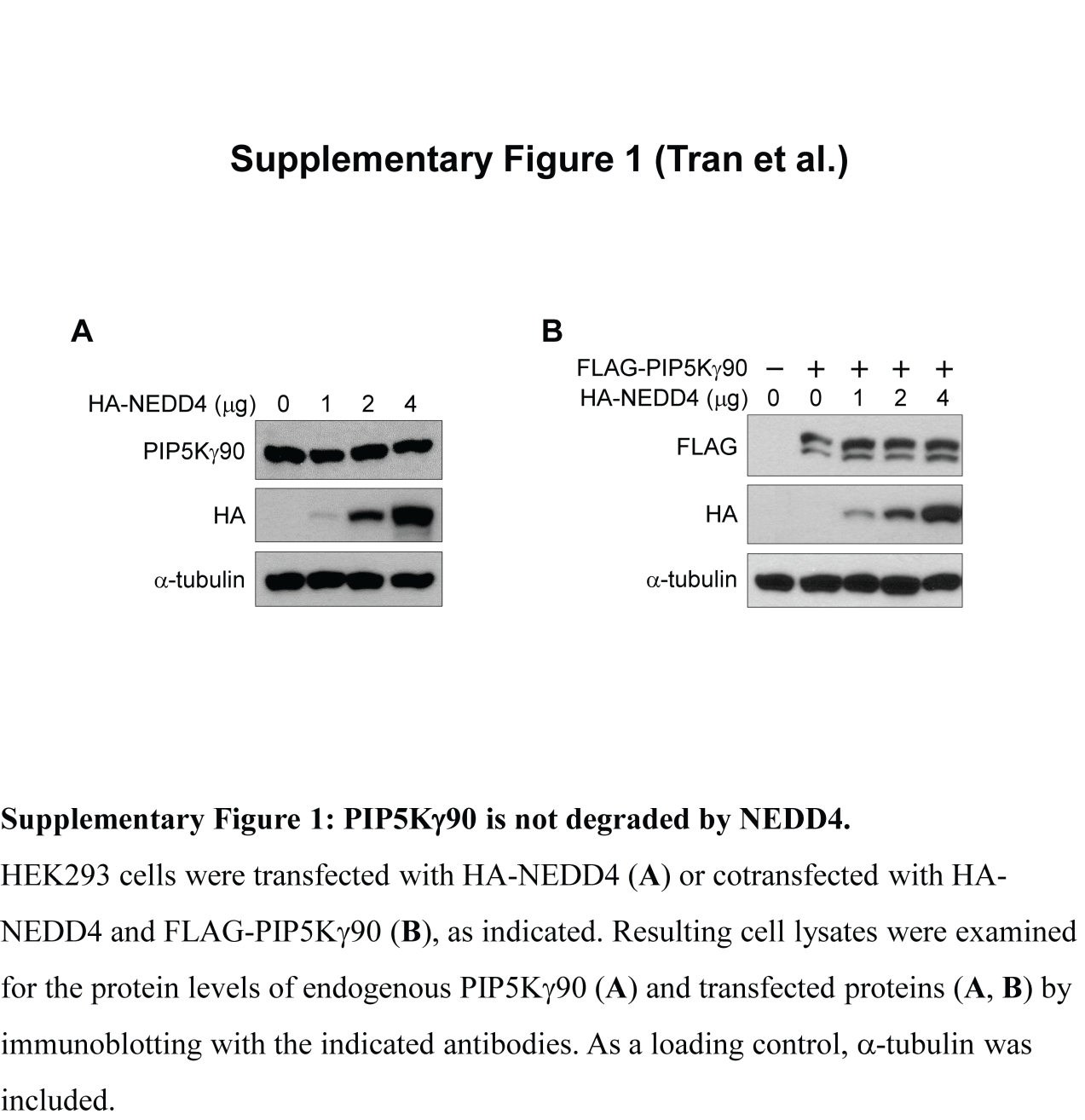


**Supplementary Fig. 3** PIP5Kγ90 is not degraded by NEDD4. HEK293 cells were transfected with HA-NEDD4 (**A**) or cotransfected with HA-NEDD4 and FLAG-PIP5Kγ90 (**B**), as indicated. Resulting cell lysates were examined for the protein levels of endogenous PIP5Kγ90 (**A**) and transfected proteins (**A**, **B**) by immunoblotting with the indicated antibodies. As a loading control, α-tubulin was included.


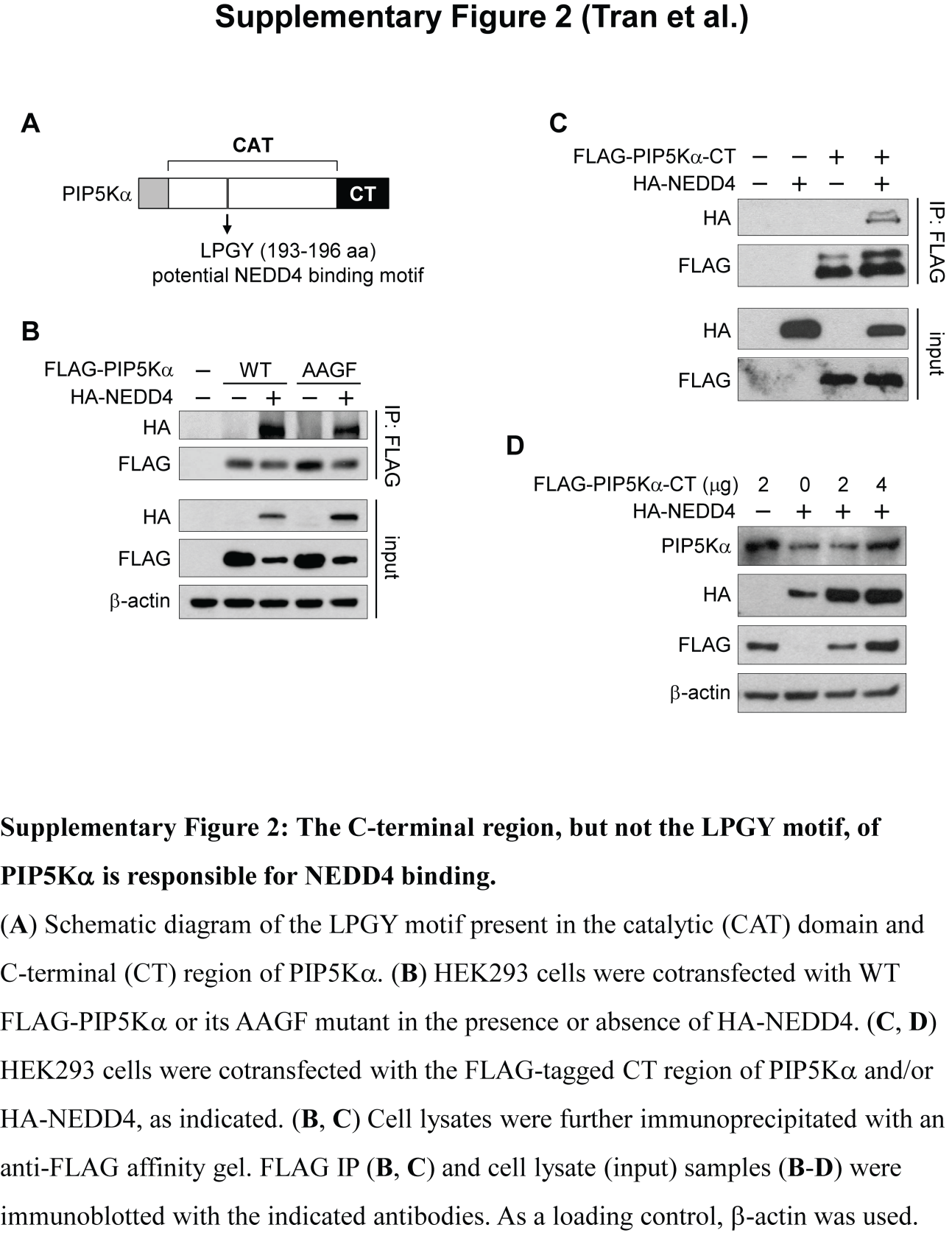


**Supplementary Fig. 4** The C-terminal region, but not the LPGY motif, of PIP5Kα is responsible for NEDD4 binding. (**A**) Schematic diagram of the LPGY motif present in the catalytic (CAT) domain and C-terminal (CT) region of PIP5Kα. (**B**) HEK293 cells were cotransfected with WT FLAG-PIP5Kα or its AAGF mutant in the presence or absence of HA-NEDD4. (**C**, **D**) HEK293 cells were cotransfected with the FLAG-tagged CT region of PIP5Kα and/or HA-NEDD4, as indicated. (**B**, **C**) Cell lysates were further immunoprecipitated with an anti-FLAG affinity gel. FLAG IP (**B**, **C**) and cell lysate (input) samples (**B**−**D**) were immunoblotted with the indicated antibodies. As a loading control, β-actin was used.


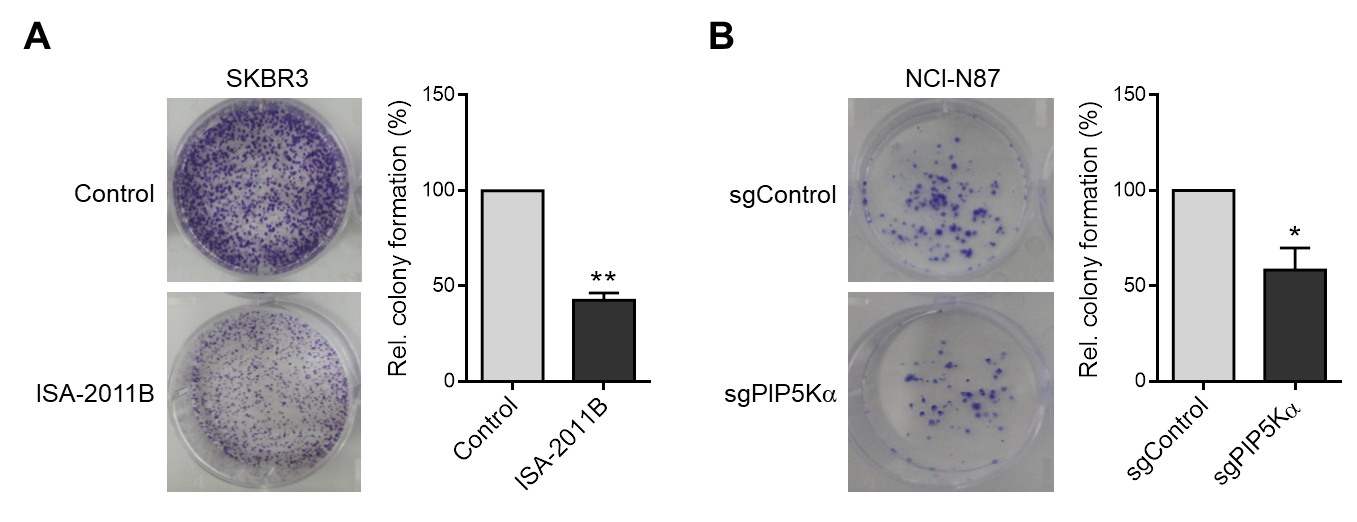


**Supplementary Fig. 5** Effect of a PIP5Kα inhibitor or its gene knockout on cancer cell proliferation. Colony formation assays were performed in SKBR3 cells treated with a PIP5Kα inhibitor ISA-2011B (20 μM) or DMSO as a control (**A**) and in control or PIP5Kα knockout (sgPIP5Kα) NCI-N87 cells (**B**). Values in the graphs are presented as the mean ± SEM. **p*<0.05, ***p*<0.01.

**Supplementary Table 1** qRT-PCR primers used in this study

| Gene | Sequences |
| --- | --- |
| E2F1 | Forward, 5'-CATCCCAGGAGGTCACTTCTG-3' Reverse, 5'-GACAACAGCGGTTCTTGCTC-3' |
| CDK1 | Forward, 5'-TTCAGAGCTTTGGGCACTC-3' Reverse, 5'-ATGCTAGGCTTCCTGGTTTC-3' |
| CCND1 | Forward, 5'-GCTGCGAAGTGGAAACCATC-3' Reverse, 5'-CCTCCTTCTGCACACATTTGAA-3' |
| FOXO3 | Forward, 5'-ACGGCTGACTGATATGGCAG-3' Reverse, 5'-CGTGATGTTATCCAGCAGGTC-3' |
| PIP5Kα | Forward, 5'-GGCACAAGTGACAACAAAGG-3'  Reverse, 5'-CAAAGGTGGAGTCTGAGGTAAA-3' |
| GAPDH | Forward, 5'-AGGGCTGCTTTTAACTCTGGT-3' Reverse, 5'-CCCCACTTGATTTTGGAGGGA-3' |
